# Protective effect of *Carica papaya* ethanolic leaf extract against lead-induced toxicity in Wistar rats

**DOI:** 10.3389/ftox.2026.1762172

**Published:** 2026-04-10

**Authors:** Richard Haakonde, Golden Zyambo, Katendi Changula, Roy Mwenechanya, Kaampwe Mayovu Muzandu

**Affiliations:** 1 Toxicology Laboratory, School of Veterinary Medicine, Department of Biomedical Sciences, University of Zambia, Lusaka, Zambia; 2 Biochemistry Laboratory, School of Veterinary Medicine, Department of Biomedical Sciences, University of Zambia, Lusaka, Zambia; 3 Histopathology Laboratory, School of Veterinary Medicine, Department of Paraclinical Studies, University of Zambia, Lusaka, Zambia

**Keywords:** Carica papaya, flavonoids, lead toxicity, oxidative Stress, Pb-acetate, Phenolics

## Abstract

**Introduction:**

Lead poisoning remains a significant public health issue globally, with varied negative health outcomes, particularly in developing countries. Lead is a pervasive environmental pollutant that induces systemic toxicity, largely through oxidative stress resulting from its tissue bioaccumulation and disruption of normal physiological processes in a biological system. Plant-derived chelators and antioxidants may provide low-cost strategies to mitigate Pb-induced damage in both humans and animals. This study was aimed at evaluating the protective effect of *Carica papaya* leaf ethanolic extract against lead-induced toxicity in Wistar rats.

**Methods:**

The protective effect of *Carica papaya* leaf ethanolic extract was evaluated in Wistar rats exposed to Pb – acetate, 50 mg/kg body weight. Thirty animals were assigned to six groups (n = 5) and treated for 30 days with vehicle – double distilled water, Pb – acetate alone, or Pb – acetate in combination with *Carica papaya* leaf ethanolic extract (50, 100, 200 and 400 mg/kg body weight). Weekly changes in body weight were monitored across all exposure groups. Lead concentrations were then quantified in blood, liver, kidney, and bone tissues. Additionally, oxidative stress and lipid peroxidation were evaluated by measuring malondialdehyde levels in liver homogenates for all treatment groups.

**Results and Discussion:**

Pb – acetate exposure led to significant Pb accumulation in blood, liver, kidney, and bone, as well as increased hepatic malondialdehyde levels (*p*<0.05). Co-administration of the extract particularly at doses ≥100 mg/kg BW, showed significant corresponding reduction of Pb accumulation in blood, liver, and kidney, and liver malondialdehyde levels. This study provides preliminary indications that *Carica papaya* leaf ethanolic extract may provide protection against Pb-induced toxicity, likely through its flavonoid and phenolic chelation and antioxidant activities, coupled with Pb tissue deposition mitigation. These findings suggest Carica papaya leaf ethanolic extract could have potential, to be a low-cost phytotherapeutic candidate for managing Pb poisoning.

## 1 Introduction

Lead (Pb) poisoning remains a significant public health issue globally, with varied negative health outcomes, particularly in developing countries. Zambia has not been spared from this problem. Kabwe district, the capital of Central Province in Zambia, has been reported to have widespread lead-contaminated soils (Bose-O’Reilly et al., 2018). A study conducted in 2022 in Kabwe town reported an average of surface soil Pb levels of 1,487 mg/kg (dry weight), a figure significantly higher than WHO guidelines for both residential and non-residential areas (Nakata et al., 2022). Soil is essential to the locals’ livelihoods as it supports their various economic activities such as farming, brick making, and mining. These activities, however, expose humans and other life forms to lead poisoning.

Health data studies also show that over 95% of children living in the most affected townships in Kabwe have high blood lead levels (BLLs) of greater than 10 μg/dL (Bose-O’Reilly et al., 2018). Approximately 50% of these children have BLLs ≥ 45 μg/dL (Bose-O’Reilly et al., 2018). Chronic exposure to lead can result in severe health problems, including neurological, haematological, and renal damage (Flora et al., 2012). In humans, Pb poisoning can lead to anemia by inhibiting ferrochelatase and δ-aminolevulinic acid dehydratase (ALAD), which are among the various enzymes essential for heme biosynthesis (Mense and Zhang, 2006). Lead’s inhibition of ferrochelatase and ALAD reduces heme production, resulting in anemia (Mense and Zhang, 2006).

Current treatments for lead poisoning such as sodium calcium edetate or ethylene diaminetetra acetate (EDTA) and succimer (Dimercaptosuccinic Acid - DMSA) used in the treatment of inorganic Pb in humans are both Pb chelators, with documented adverse side effects; sodium calcium edetate causes dose-related nephrotoxicity (Bradberry et al., 2009). Both chelators cause depletion of zinc and copper, with EDTA having a significantly higher zinc depletion effect than succimer (Bradberry et al., 2009). Another study by Bradberry et al. (2009) showed that in as much as DMSA is not teratogenic, however it did produce maternal toxicity (decreased weight gain) and fetotoxicity in high doses (100–1,000 mg/kg/day) in rats. Most synthetic chelators lack direct inherent antioxidant properties to directly scavenge reactive oxygen species (ROS), and reactive nitrogen species (RNS) generated under oxidative and nitrosative stress (Chandimali et al., 2025; Chaudhary et al., 2023). Additionally, they do not directly stimulate the upregulation of key antioxidant enzymes such as catalase (CAT) and superoxide dismutase (SOD), nor do they directly enhance the synthesis of endogenous metal-binding proteins like metallothionein (Kuo et al., 2001).

The need to explore natural remedies, such as plant extracts, including *Carica papaya* leaf extract (CPLE), as a potentially cost-effective alternative treatment option is well justified. Several ethnomedical products have been demonstrated to possess medicinal effects against several pathogenic infections, inflammatory diseases, and poisoning caused by both organic and inorganic compounds other than heavy metals. Investigations of these products for their anti-inflammatory and anti-bioaccumulation properties against heavy metals including Pb in biological systems is important, despite not being extensively studied. These products are cheap and readily available in communities including those with limited resources. *Carica papaya*, commonly known as *papaya*, a member of the *Caricaceae* family, is known to contain a diverse array of compounds and biomolecules, notably papain, flavonoids, phenolics and alkaloids, which have immense industrial and medicinal value (Babalola et al., 2023).

Kong et al. (2021) demonstrated that CPL extracts contain high anti-inflammatory, antioxidant, anti-ulcer, and larvicidal components such as flavonoids, and phenolic compounds. Flavonoids have been shown to act as antioxidants and chelating agents, hence used for the management of heavy metal toxicity (Dewanjee et al., 2013; Francis et al., 2020; Guo et al., 2024; Heim et al., 2002; Liu et al., 2013; 2010; Okon et al., 2023; Sharma et al., 2020). Chelators bind to metals including Pb and help eliminate them from the body *via* urine, however most of these chelators do not act as anti-inflammatory or antioxidant agents (Thuppil and Tannir, 2013). Notable among the flavonoids contained in ethanolic CPLE is quercetin, which is a known strong antioxidant and metal chelator shown to have protective effects against Pb-induced toxicity (Arslan et al., 2022).

Given the global burden of lead toxicity and the need for accessible treatment options, this study seeks to validate the use of *Carica papaya* leaf extract as a potential therapeutic agent. This could significantly impact public health, particularly in regions with high lead exposure. Exploring the protective effects of CPLE could provide a viable, low-cost alternative for treating lead poisoning not only in Zambia but the world at large. This approach leverages a locally available resource, making it particularly suitable for resource-limited settings. Public health interventions, such as incorporating CPLE as additives in food and drinks, can help mitigate the long-term effects of lead toxicity, including neurological impairments in children, as well as liver and kidney cancers. Partnering with manufacturers to introduce these products in areas heavily affected by lead exposure could be a key strategy in reversing the damage caused by lead poisoning. Concisely, this study is aimed at evaluating the protective effect of *C. papaya* ethanolic leaf extract against lead-induced toxicity in Wistar rats.

## 2 Materials and methods

### 2.1 Chemicals and reagents

Hydrogen peroxide (30%), sodium carbonate, sodium nitrite, aluminum chloride, lead (II) acetate trihydrate, and lead standard solution (Pb-1000) for atomic absorption spectrometry were obtained from Kanto Chemical Co., Inc. (Tokyo, Japan). Nitric acid (69%), ethanol (95%), and isoflurane were purchased from HiMedia Laboratories Pvt. Ltd. (Mumbai, India), Xilong Scientific Co., Ltd. (Guangdong, China), and FUJIFILM Wako Pure Chemical Corporation (Osaka, Japan), respectively. Folin–Ciocalteu’s phenol reagent (2.0 N) and gallic acid (3,4,5-trihydroxybenzoic acid) were procured from Oxford Lab Fine Chem LLP (Maharashtra, India). The malondialdehyde (MDA) assay kit (Lot no. 2503003001) and quercetin (≥95%, 2-(3,4-dihydroxyphenyl)-3,5,7-trihydroxy-4H-1-benzopyran-4-one) were purchased from Beijing Solarbio Science and Technology Co., Ltd. (Beijing, China) and Sigma-Aldrich (St. Louis, United States), respectively. All chemicals and reagents used were of analytical grade.

### 2.2 Preparation of ethanolic CPLE

Healthy, fresh, and mature leaves of *Carica papaya* were collected on 5 June 2025 from Meanwood Ndeke, Chongwe District (15° 18′7.65″S, 28° 24′59.37″E) in Lusaka Province, Zambia. The plant was authenticated by the taxonomists at the University of Zambia, School of Natural Sciences, Department of Biological Sciences, Great East Road Campus in Lusaka, Zambia. A voucher specimen was deposited at the department’s herbarium for future reference.

The collected leaves were thoroughly washed with tap water, rinsed with distilled water, and air-dried. The veins were removed, and the leaves were shed-dried for 72 h at 25 C ± 2 °C. The dried material was then ground into a fine powder using a laboratory blender. A 500 g portion of the powdered sample was extracted with 1.5 L of 70% ethanol using the maceration method. During extraction, the mixture was intermittently stirred at 700 revolutions per minute (rpm) using a magnetic stirrer equipped with Teflon-coated stir bars to ensure uniform mixing. After 72 h, the mixture was filtered under vacuum using Whatman No. 1 filter paper and a Büchner flask. The filtrate was concentrated under reduced pressure and subsequently freeze-dried to obtain the lyophilized ethanolic extract as illustrated in Supplementary Figure S1.

The final extract was stored at −20 °C until use. For animal studies, the ethanolic extract of *Carica papaya* as reconstituted in ultrapure double-distilled water (DDW) to the desired concentrations for oral administration. The dosing regimen for the ethanolic CPLE was adapted from Francis et al. (2020). The extract preparation procedure was modified from the method described by Francis et al. (2020).

The percentage yield of the extract was calculated using the following formula as described by Francis et al. (2020):

$$\text{Percentage Yield }\left(\%\right)=\frac{\left(\text{Weight}\;\text{of}\;\text{Original}\;\text{Extract}\right)}{\left(\text{Weight}\;\text{of}\;\text{the}\;\text{Powder}\right)}\times 100$$

### 2.3 Determination of total phenolic content

The total phenolic content (TPC) of the CPLE was measured using the Folin–Ciocalteu colorimetric assay, adapted to the procedure described by Khamphaya et al. (2022), with slight modifications to enhance reagent ratios and reaction stability for this sample matrix. CPLE was reconstituted in double-distilled water (DDW) to give a final concentration of 1 mg/L. A 250 μL portion of this solution was transferred to a microcentrifuge tube and mixed with 250 μL of 10% (v/v) Folin–Ciocalteu reagent. The mixture was briefly vortexed to achieve total homogeneity and subsequently allowed to stand for 5 min at room temperature (25 °C ± 2 °C) to promote initial complex formation between phenolic compounds and the reagent.

Further to the pre-reaction period, 500 μL of freshly produced 7.5% (w/v) sodium carbonate (Na_2_CO_3_) was added to the reaction mixture to develop the alkaline conditions required for chromophore formation. The total reaction volume was subsequently adjusted to 1.5 mL with deionized distilled water. Samples were thoroughly mixed and then incubated for 90 min in the dark at room temperature to reduce the risk of light-induced degradation of the chromophores as well as facilitate complete color development.

After incubation, 200 μL of each reaction mixture was transferred to the 96-well microplate. Absorbances were measured at 765 nm with a Thermo Scientific 650 Multiskan FC Microplate Reader. Gallic acid reconstituted in double deionized water was used as the calibration standard, with a concentration range of 0–200 mg/L prepared to create a standard curve for quantification purposes. All measurements were conducted in duplicate to assess technical repeatability, and mean absorbance values were utilized for further calculations of TPC.

### 2.4 Determination of total flavonoid content

The total flavonoid content (TFC) of the CPLE was determined using the aluminum chloride (AlCl_3_) colorimetric method, following the procedure proposed by Khamphaya et al. (2022), with some modifications to increase consistency and analytical sensitivity. 50 μL of CPLE (1 mg/L) was reconstituted in 70% ethanol for complete dissolution of the constituents. The reconstituted sample was then diluted 1:1 with 70% ethanol to achieve a final working volume of 100 µL. The dilution step was done to reduce matrix interferences and ensure absorbance remains within the linear detection range.

To initiate complex formation, 300 µL of a freshly made 10% (w/v) AlCl_3_ solution was added to each sample tube. The reaction mixture was gently mixed to promote consistent interaction between the aluminum ions and the flavonoid molecules, then left to stand for 6 min at ambient laboratory temperature. The incubation period enhances the development of stable Al^3+^–flavonoid complexes, which contribute to the distinctive yellow color measured at 510 nm. Further to the initial reaction phase, 300 µL of a 5% (w/v) sodium nitrite (NaNO_2_) solution was added. The mixture was briefly vortexed to ensure complete mixing and then incubated for an additional 15 min. Sodium nitrite promotes the diazotization reaction, which promotes chromophore formation and thus increases test sensitivity.

After completion of the reaction, a 200 µL aliquot of the final mixture was transferred to a 96-well microplate for spectrophotometric analysis. Absorbances were measured at 510 nm with a Thermo Scientific 650 Multiskan FC Microplate Reader.

Quercetin, reconstituted in 70% ethanol, was used as the reference standard. A calibration curve was developed within a concentration range of 0–100 mg/L, enabling the quantification of sample flavonoid content in quercetin equivalents (mg QEC/L). All measurements were performed in duplicate to evaluate analytical reproducibility and minimize variability. The entire technique was conducted under regulated laboratory conditions to minimize potential environmental factors, such as temperature variations and light exposure, which may impact chromophore stability.

### 2.5 Pb-acetate preparation and plant extract (CPLE) dosing

Lead (II) acetate trihydrate [(CH_3_COO)_2_ Pb.3H_2_O] (PbAc) was obtained from Kanto Chemical Co., Inc. (Tokyo, Japan). A dose of 50 mg/kg body weight (BW) of PbAc was used to induce toxicity in Wistar rats, based on previous reports demonstrating that oral administration of Pb at doses up to 60 mg/kg over 8 weeks induces oxidative stress and tissue damage without mortality (Khamphaya et al., 2022; Kumar Singh et al., 2018).

A stock solution of PbAc (125 mg/mL) was freshly prepared each day by dissolving 2.5 g of Pb-acetate in 20 mL of ultrapure DDW. The appropriate volume for each rat was calculated based on its body weight and the target dosage (50 mg/kg BW) and administered orally by gavage. The Pb-acetate dose (50 mg/kg BW) was chosen to model chronic lead toxicity by inducing sustained internal lead accumulation and consistent toxicological effects in a sub-chronic animal model (Khamphaya et al., 2022). Because of interspecies variability in toxicokinetic and experimental time limits, this administered dose does not replicate human or animal environmental exposure levels but is widely used to simulate chronic lead burden in experimental settings.

Previous studies have shown that oral administration of CPLE at doses up to 2,000 mg/kg BW in rats for up to 28 days and, in some cases, up to 13 weeks, caused no morbidity or mortality (Francis et al., 2020; Halim et al., 2011; Rani et al., 2023; Taychaworaditsakul et al., 2024). In this study, CPLE dosages ranging from 50 to 400 mg/kg BW were used.

### 2.6 Study site

The study was conducted at the University of Zambia, School of Veterinary Medicine, Department of Biomedical Sciences.

### 2.7 Sample size determination

The sample size (n) was calculated using the formula described by Gogtay (2010):

$$n=\frac{{\left(Z_{\alpha }+Z_{\beta }\right)}^{2}.\left(k-1\right).\sigma^{2}}{\Delta^{2}}$$

Where:

- *σ* = standard deviation of the outcome = 5
- (1-*β*) = desired power = 0.8 (Z_*β*_ ≈ 0.84).
- *α* = significance level = 0.05 (Z_*α*_ ≈ 1.96).
- *K* = number of groups = 6
- Δ = minimum detectable difference (effect size) = 14

Substituting the values:

$$n=\frac{{\left(1.96+0.84\right)}^{2}\left(6-1\right){\left(5\right)}^{2}}{{\left(14\right)}^{2}}=\frac{{\left(2.8\right)}^{2}.\left(5\right).25}{196}$$

n≈5

To find differences between six groups, a power analysis was used to determine the sample size. The calculation was predicated on a statistical power of 80% and a two-tailed significance level of 0.05. Based on prior data, the outcome’s standard deviation was calculated to be 5. The smallest biologically significant change between groups was represented by the minimum detectable difference (Δ), which was set at 14 units. Five animals per group was the necessary sample size based on these presumptions. Thus, the study comprised a total of 30 rats (six groups of five rats each).

### 2.8 Experimental design

A cross-sectional experimental study design using a rat model was employed, were thirty healthy male Wistar rats were randomly divided into six groups of five animals per treatment group (n = 5 per group). The 30-day exposure period, dose selection and co-administration of PbAc and CPLE were based on previously published studies (Francis et al., 2020; Kataba et al., 2021; Khamphaya et al., 2022; Liu et al., 2013; Ukwa et al., 2025). All treatments were administered orally via gavage.

- Group 1 (NC–Normal Control): The animals received double distilled water as a vehicle for the entire study period.
- Group 2 (PC–Positive Control): The animals in this group received 50 mg/kg body weight of PbAc only throughout the study period.
- Group 3 (LD–Low Dose): This group was treated with 50 mg/kg body weight PbAc and 50 mg/kg BW CPLE co-administration.
- Group 4 (MD–Medium Dose): This group was treated with 50 mg/kg body weight PbAc and 100 mg/kg BW CPLE co-administration.
- Group 5 (HD–High Dose): This group was treated with 50 mg/kg body weight PbAc and 200 mg/kg body weight CPLE co-administration.
- Group 6 (VHD–Very High Dose): This group was treated with 50 mg/kg BW PbAc and 400 mg/kg body weight CPLE co-administration.

#### 2.8.1 Experimental animals

Healthy male Wistar rats 6 weeks old, with weights ranging between 100 g and 250 g were sourced from the Small Animals Breeding Section, Central Services and Supply Unit, University of Zambia, School of Veterinary Medicine. The animals were housed under standard laboratory conditions (12-h light/dark cycle, 25 °C ± 2 °C, 50 ± 15% relative humidity) and provided with a standard diet and water *ad libitum*.

All experimental procedures were conducted in accordance with institutional and international guidelines for the ethical use of animals in research. Handling and experimental procedures were performed by trained personnel from the School of Veterinary Medicine, UNZA. Humane endpoints were established, and animals showing signs of severe or unrelieved distress were euthanized using isoflurane. Animal use was minimized through proper research design and statistical planning in adherence to the 3Rs principle (Replacement, Reduction, and Refinement).

Prior to the experiment, animals were acclimatized for 7 days and randomly assigned to six groups n = 5 per group based on the prior sample size determination. Additionally, the body weights of the rats were recorded on day one and thereafter, weekly, throughout the experimental period.

### 2.9 Weekly monitoring of body weight

Body weight measurements were taken for all experimental animals at the beginning of the study (day 1) and then at weekly intervals during the exposure period, for a total of five recorded measurements per animal. Regular body weight monitoring provided a non-invasive and sensitive indicator of physiological wellbeing, allowing for the detection of minor changes in growth patterns, metabolic condition, and overall health. This measure is especially important for toxicological investigations, as heavy metals like lead (Pb) are known to interfere with nutrition absorption, disrupt endocrine signaling, and induce metabolic stress, each of which may result in reduced or fluctuating weight gain. Conversely, the assessment allowed for an evaluation of the possible protective or restorative benefits of CPLE in ameliorating Pb-induced toxicity. By comparing weekly weight changes across treatment groups, the study was aimed at finding whether CPLE could promote normal development patterns or prevent weight loss resulting from Pb exposure. This analytical approach ensured a valid dataset for assessing treatment-related physiological changes and helped to assess overall animal health and research outcomes.

### 2.10 Sample collection

All laboratory materials, including glassware, plasticware, and dissection instruments, underwent thorough decontamination before use to reduce the risk of trace metal interference. Decontamination required placing of the materials in 2% nitric acid for at least 12 h, followed by thorough rinsing with double deionized water to remove residual acid as well as possible contaminants. The items were subsequently dried in a ventilated oven at 50 °C to ensure the complete removal of moisture prior to contact with any biological samples.

Following the 30-day exposure period, the animals underwent an overnight fast to minimize variability in metabolic markers and circulating lipids. The next morning, they were moved to the dissection room, which was maintained under controlled environmental conditions. Animals were euthanized with inhaled 5% isoflurane, in accordance with the study by Marquardt et al. (2018). Anesthesia was induced and maintained at a surgical level, as demonstrated by the absence of a pedal withdrawal reflex prior to euthanasia.

Blood, liver, kidney, and femur (bone) samples were collected for quantitative metal analysis and subsequent biochemical assays. Organs were excised with acid-washed instruments, gently rinsed with ice-cold normal saline to eliminate excess blood and surface contaminants and blotted dry on sterile absorbent tissue to standardize moisture content in the samples. Approximately 0.5 g tissue portions from each liver, aimed for lipid peroxidation assessment (MDA analysis), were placed directly into pre-labeled cryovials containing 500 µL of ice-cold 0.1 M Tris–HCl–0.001 M EDTA buffer (pH 7.4). The immediate immersion in buffer reduced enzymatic degradation and maintained oxidative stress markers. Aliquots were snap-frozen and subsequently stored at −80 °C for biochemical analysis. The remaining biological samples, which included whole blood, kidneys, bone, and residual liver tissue, were stored at −20 °C to ensure sample stability for metal quantification and additional assays. Together with animal tissues, representative samples related to the environment and exposure were collected. The materials used consisted of commercial feed pellets, distilled water provided to the animals, C. papaya leaves used during the exposure protocol, and double deionized water used in all laboratory procedures. The materials underwent processing to ascertain background and exposure-related lead concentrations, facilitating the evaluation of potential contamination sources and the validation of the exposure model. Sample analyses were performed at the Toxicology Laboratory within the Department of Biomedical Sciences at the School of Veterinary Medicine, University of Zambia, Great East Road Campus. The laboratory follows established quality-assurance procedures, with all analytical procedures—from sample digestion to instrumental metal quantification—conducted according to standard operating protocols to ensure the accuracy, reproducibility, and reliability of the data produced.

### 2.11 Determination of Pb concentration

Lead concentrations in blood, liver, kidney, and femoral bone were measured using a modified analytical approach based on Kataba et al. (2021), aimed at enhancing digestive efficiency, matrix decomposition, and analytical consistency. For each of the samples, 150–300 mg of solid tissue (liver, kidney, and bone) and 100 μL of whole blood were set aside for analysis. Tissue samples were initially dried in an oven at 50 °C for 48 h to attain a consistent mass and minimize variability related to moisture content. After dehydration, the liver, kidney, and bone were transferred into acid-cleaned Teflon digestion tubes for chemical digestion.

A closed microwave digesting system (SpeedWave MWS-2; Berghof, Eningen, Germany) was used to mineralize all biological matrices under regulated temperature and pressure conditions. Each sample was treated with 5 mL of 30% trace-metal-grade nitric acid (HiMedia Laboratories Pvt. Ltd., Mumbai, India) and 1 mL of 30% hydrogen peroxide (Kanto Chemical Co., Inc., Tokyo, Japan). The microwave program parameters, consisting of temperature ramp rates, hold durations, and maximum pressures, are shown in Supplementary Table S1 and were selected to ensure complete oxidation of organic materials while avoiding analyte volatilization.

After digestion, vessels were allowed to cool to room temperature prior to opening to prevent abrupt pressure release. Digestates were quantitatively transferred to 15 mL polypropylene tubes, and ultrapure water produced by a Milli-Q purification system (Merk, Darmstadt, Germany) was added to give a final volume of 10 mL of the digested sample. To reduce the risk of cross-contamination, all digestion vessels were subjected to a decontamination process using 60% nitric acid after each digestion cycle. Lead analysis was carried out at a wavelength of 283.3 nm using a Polarized Zeeman Atomic Absorption Spectrophotometer (AAS) (Model Z-2010; Serial No. 2137-009; Hitachi High-Technologies Corporation, Tokyo, Japan). Instrumental parameters, among which are lamp current, slit width, burner setup, and background correction settings, are specified in Supplementary Table S2. Calibration curves were generated from certified lead standards, and instrument stability was assessed through regular measurements of calibration verification solutions.

To rule out possible external sources of Pb exposure, the same digestion and analytical methodologies were applied for feed pellets, distilled water, Carica papaya leaves, and the double deionized water provided to the experimental animals, except that water samples were analyzed without digestion owing to their pre-existing inorganic matrix. This concurrent study facilitated the evaluation of potential environmental or dietary contaminants that could influence Pb accumulation in tissues. Supplementary Figure S2 illustrates the workflow involved in the sample processing for Pb quantification.

Quality control protocols were carried out with matrix-specific certified reference materials. DOLT-5 (dogfish liver; National Research Council, Canada) was utilized for liver and kidney analysis; Seronorm (SERO AS, Norway) for blood; and Bone Ash (SRM 1400; Monsanto Co., United States) for bone assessment. The reference materials were processed and evaluated under identical conditions as the experimental samples. The recovery rates obtained were 117.90% for DOLT-5, 117.65% for Seronorm, and 80.82% for Bone Ash. While recoveries beyond 100% may suggest matrix-induced enhancement, the values were below acceptable limits for trace-metal tests, indicating adequate digestion completeness and analytical precision. The technique detection limits were 0.0003 mg/L for blood, 0.00128 mg/L for liver, 0.00006 mg/L for kidney, and 0.00016 mg/L for bone, ensuring sufficient sensitivity for detecting Pb quantities anticipated in biological matrices from animals exposed to environmental factors.

### 2.12 Lipid peroxidation determination (MDA assay)

The method used for the analysis of MDA in rat liver homogenates was adapted from the MDA assay kit insert, Lot no. 2503003001; Beijing Solarbio Science and Technology Co., Ltd., China, with some modifications. Liver samples, earlier stored at −80 °C in 500 µL of 0.1 M Tris–HCl–0.001 M EDTA buffer (pH 7.4), were thawed at a controlled room temperature of 25 °C ± 2 °C. Afterwards, 0.1 g of tissue from each sample was placed into 2 mL Teflon self-standing tubes containing 1 mL of ice-cold extraction solution provided with the MDA test kit (Lot No. 2503003001; Beijing Solarbio Science and Technology Co., Ltd., China). To improve mechanical disruption efficiency, six zirconia (ZrO_2_) beads were incorporated within each tube. Homogenization was carried out with a Taitec Beads Crusher μ-12 (SN 80300482) at 1,800 rpm for 60 s, a parameter selected to provide uniform cell lysis while preventing excessive tissue heating. After homogenization, samples were centrifuged at 8,000 *g* for 10 min at 4 °C to precipitate insoluble debris. The resultant supernatant was immediately placed into pre-chilled 2 mL Eppendorf tubes and kept on ice to minimize any post-extraction oxidative alterations. The reaction mixtures for the thiobarbituric acid (TBA) assay were prepared in accordance with the manufacturer’s guidelines, as detailed in Supplementary Table S3.

Each reaction tube was sealed and placed in a boiling water bath at 100 °C for 60 min. The high-temperature incubation facilitates the synthesis of the MDA–TBA compound, which demonstrates significant absorbance at 532 nm. Safety measures, such as the use of heat-resistant tube caps and protective shielding, were used to prevent splashing or sample loss during boiling.

After the initial incubation, the tubes were immediately chilled in an ice bath to terminate the reaction and inhibit additional compound formation. Subsequently, samples were further centrifuged at 10,000 × *g* for 10 min at room temperature to clear up the sample mixtures and eliminate precipitated proteins. 200 μL of the resultant supernatant was transferred to a 96-well microplate, ensuring that all samples and standards were put in duplicate to reduce analytical variability. The Thermo Scientific 650 Multiskan FC Microplate Reader was preheated for 35 min to stabilize the optical system for spectrophotometric analysis. The device was calibrated with distilled water before sample loading. Absorbance at 532 nm was immediately measured. Supplementary Figure S3 shows the workflow used in the MDA assay in this present study.

MDA concentrations were determined using Beer–Lambert’s law. The molar extinction coefficient for the MDA–TBA complex (ε = 1.55 × 10^5^ L mol^-1^ cm^-1^ at 532 nm) and the optical path length of the 96-well plate (d = 0.6 cm) were used to convert absorbance (ΔA_532_) into molar concentration. The sample weight (W = 0.1 g), total reaction volume (V total = 5 × 10^−4^ L), and dilution factor (F = 1) were incorporated in the final calculations. The formula used was:

$$\text{MDA Content }\left(\text{nmol}/g\right)=\left[\Delta A532\times V_{\text{total}}÷\left(\varepsilon \;x\;d\right)\times 10^{9}\right]÷W*F$$

Where:

ε = MDA molar absorption coefficient (1.55 × 10^5^/mol × cm at 532 nm), d = optical path of 96-well plate (0.6 cm), W = mass of sample in g (0.1 g), V_total_ = 5 × 10^−4^ L, and F = dilution factor (1).

Substitution of the constants gives:

$$\text{MDA Content }\left(\text{nmol}/g\right)=53.763\times 0.067/W*F.$$

The application of this computational method guarantees a quantitative evaluation of lipid peroxidation in hepatic tissue and facilitates direct comparison among experimental groups.

### 2.13 Data analysis

Data analysis was conducted using GraphPad Prism software (Version 10.5.0; GraphPad Software, San Diego, CA, United States). All quantitative results were expressed as means ± standard deviation (SD). Prior to carrying out group comparisons, the underlying assumptions for parametric testing were assessed. The Shapiro–Wilk test was utilized to evaluate the normality of each variable’s distribution, whereas the Brown–Forsythe test was used to evaluate the homogeneity of variances among groups. Variables meeting both assumptions (*p* > 0.05 for normality and homogeneity of variance) were subjected to one-way analysis of variance (ANOVA), then followed by Tukey’s *post hoc* test to determine pairwise differences among treatment groups.

Welch’s ANOVA was used as a more robust alternative for datasets where the assumptions of normality or homogeneity of variance were violated (*p* < 0.05). Post hoc comparisons for these analyses were performed using Dunnett’s T3 test, which does not assume equal variances and ensures accurate control of type I error in heteroscedastic situations. A two-way ANOVA was used to evaluate longitudinal changes in weekly body weight of Wistar rats, with time and treatment as fixed factors. Tukey’s multiple comparison test was applied to assess main effects and interactions.

Statistical significance was set at p < 0.05. In graphical representations, notable variations across treatment groups were denoted by unique lowercase letters (a, b, c, …), facilitating visual differentiation of groups that differed statistically according to the applied *post hoc* criteria.

### 2.14 Ethical considerations

Ethical approval for this study was obtained from the Excellence in Research Ethics and Science (ERES) Converge Zambia, under approval number 2025-May-042. The study was carried out in strict compliance with established national and international regulations on the care and utilization of animal subjects in scientific research.

## 3 Results

The study findings comprised the, determination of the percentage yield of CPLE; quantification of phytochemical constituents, (TFC and TPC) in the CPLE; weekly monitoring of body weight in the experimental animals; determination of Pb concentrations in various tissues (blood, liver, kidney and bone) and its quality assurance data consisting of AAS (IDL and IQL data, certified reference materials recovery rates, and determination of Pb levels in study materials, i.e., DW, DDW, feed, and *Carica papaya* leaves, and quantification of lipid peroxidation via MDA assay. The results for this present study were recorded as follows:

### 3.1 Determination of the percentage yield of CPLE

From the 500 g of the *Carica papaya* leaf powder that was mixed with 1.5 L of 70% ethanol for the extraction process, a yield of 102 g of crude extract was realized resulting in a percentage yield of 20.4% (w/w) as calculated below:

$$\text{Percentage Yield }\left(\%\right)=\frac{\left(\text{Weight}\;\text{of}\;\text{Original}\;\text{Extract}\right)}{\left(\text{Weight}\;\text{of}\;\text{the}\;\text{Powder}\right)}\times 100$$

= 102 g × 100.

500 g.

= 20.4%

A 20.4% (w/w) recovery was obtained from the ethanolic extraction of 500 g of starting material into 102 g of crude extract from Carica papaya leaf powder.

### 3.2 Quantification of phytochemical constituents (TFC and TPC) in the CPLE

#### 3.2.1 Determination of TPC in CPLE

As shown in Supplementary Table S4 and Table 1, and Supplementary Figure S3, the Folin–Ciocalteu method used for quantifying phenolic constituents gave a linear calibration curve for gallic acid, with (*R* ^2^ = 0.9940). The absorbance measurements from CPLE solutions duplicates gave gallic acid equivalent concentrations of 735.40 mg/L and 759.40 mg/L, thus, giving 747.40 ± 16.97 mg GEC/L adjusted mean TPC.

**TABLE 1:** Average TPC expressed as gallic acid equivalent concentration (GEC).

Sample (replicates)	Abs 1	Abs 2	Abs 3	Mean absorbance	Cal. Conc	Dil. factor	Conc GEC	Mean QEC/SD (mg/L)
1	0.373	0.349	0.355	0.359	735.40	1	735.40	747.40 ± 16.97
2	0.373	0.369	0.371	0.371	759.40	1	759.40	

Spectrophotometric absorbance measurements and subsequent determination of the average TPC in the CPLE using standard curve in Supplementary Figure S3. Average TPC was found to be 747.40 + 16.97 GEC mg/L.

#### 3.2.2 Determination of TFC in CPLE

As shown in Supplementary Table S5 and Table 2, and Supplementary Figure S5, the AlCl_3_ colorimetric method of quantifying flavonoids produced a strong linear calibration curve for quercetin (*R* ^2^ = 0.9989). The absorbance measurements corresponded to quercetin equivalent concentrations of 178.39 mg/L and 177.33 mg/L, giving a mean of 177.86 ± 0.75 mg QEC/L.

**TABLE 2:** Average TFC expressed as quercetin equivalent concentration (QEC).

Sample (replicates)	Abs 1	Abs 2	Abs 3	Mean absorbance	Cal. Conc	Dil. factor	Conc QEC	Mean QEC/SD (gm/L)
1	0.184	0.170	0.152	0.169	89.193	2	178.39	177.86 ± 0.75
2	0.168	0.162	0.173	0.168	88.667	2	177.33	

Table 2 shows spectrophotometric absorbance measurements and subsequent determination of TFC, in the CPLE, using standard curve in Supplementary Figure S4. TFC was found to be 177.86 + 0.75 QEC mg/L.

Flavonoid levels were lower than phenolic levels, however no major fluctuations were observed across replicates for the two phytochemicals. The mg/L units were used in the quantification of both TPC and TFC phytochemicals, as the extract was administered orally in liquid form, to the Pb-exposed animals.

### 3.3 Weekly monitoring of body weight in the experimental animals

Supplementary Table S6 shows the experimental animal’s body weights monitored on a weekly basis across all treatment groups throughout the 30 – day exposure period. The normal control (NC) group maintained consistent weights throughout the investigation, fluctuating within a narrow range (195.1 ± 22.77 g to 200.5 ± 22.23 g). The positive control (PC) group demonstrated a modest decrease following Week 1, with weights decreasing from 195.6 ± 30.9 g to 188.2 ± 29.29 g by Week 3, before returning to 195.2 ± 34.09 g in Week 5.

Rats in the low-dose CPLE group (LD) consistently exhibited lower mean body weights compared to the NC group across all weeks, with measurements ranging from 177.8 ± 24.92 g in Week 1 to 162.5 ± 26.41 g in Week 3, followed by a slight increase to 172.4 ± 16.92 g in Week 5. The medium-dose group (MD) displayed greater weights relative to the low-dose group (LD) and showed minimal weekly variation, maintaining weights within the range of 207.2 ± 28.15 g to 214.3 ± 36.17 g.

The high-dose (HD) and very-high-dose (VHD) groups demonstrated consistent weight trends comparable to those of the MD group. The HD group demonstrated weights ranging from 212.5 ± 21.10 g in Week 1 to 201.2 ± 18.23 g in Week 5. In contrast, the VHD group showed weights ranging from 210.7 ± 29.26 g in Week 1 to 206.1 ± 33.07 g in Week 5. No notable weekly fluctuations were observed within these categories.

Throughout the duration of the study, the LD group consistently demonstrated the lowest average body weight among all treatment groups, whereas the MD, HD, and VHD groups-maintained weights that were comparable to or marginally higher than the NC values. No group demonstrated notable weekly fluctuations in weight, and the trends remained consistent across all treatment groups.

**FIGURE 1:**
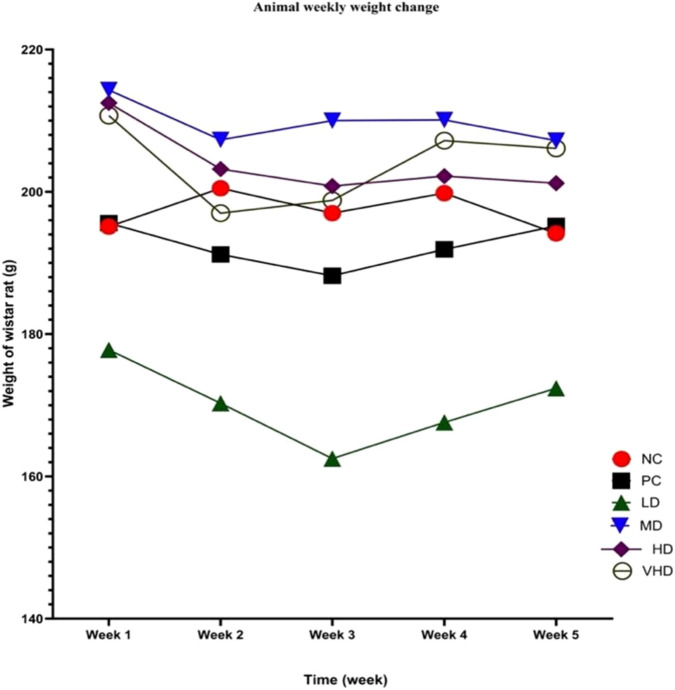
Weekly variations in body weight among mice across several treatment groups throughout a 5-week period. Body weight was documented weekly for six groups: Normal Control (NC), Positive Control (PC), Low-Dose (LD), Medium-Dose (MD), High-Dose (HD), and Very-High-Dose (VHD). Data are presented as mean ± SEM. The LD group shows a steady decrease compared to the control group, whereas the MD, HD, and VHD groups maintain relatively greater and constant weights across the research period. This graph depicts the temporal fluctuations in weight responses for each dosing regimen.

Figure 1 depicts graphically the changes of rat body weights in exposed and control groups in a 30-day study period (mean; n = 5). When tested by a two-way ANOVA and applying a Tukey’s multiple comparisons test, rat body weight changes showed a general weight loss between weeks 1 and 2 in PC and all the PbAc + CPLE exposure groups. However, an average weight gain was recorded in the NC group during the same period. PC, LD, and HD groups continued the weight loss trajectory into week 3, while both MD and VHD groups showed an average weight gain in the same week. NC control group recorded a slight weight loss in week 3. Both PC, and LD groups showed a general average weight gain in weeks 4 and 5, while NC, MD, HD, VHD showed weight loss within the same weeks. Significant weight losses (*p* < 0.05) were recorded in the MD and HD exposure groups between weeks 1 and 2 (*p* < 0.0076), and weeks 1 and 3 (*p* < 0.026) respectively. Significant weight gain was however recorded in VHD exposure group between weeks 3 and 4 (*p* < 0.035).

### 3.4 Determination of Pb concentrations in various tissues (blood, liver, kidney and bone)

Supplementary Table S7 presents a summary of lead concentrations in blood, liver, kidney, and bone across all experimental groups. The normal control (NC) group demonstrated minimal lead (Pb) concentrations across all matrices. The positive control (PC) group exhibited substantially elevated levels of Pb in blood (30.78 ± 0.61 μg/dL), liver (2.04 ± 0.07 mg/kg), kidney (3.22 ± 0.06 mg/kg), and bone (27.07 ± 1.33 mg/kg), with all values markedly exceeding those observed in the normal control (NC) group (*p* < 0.01).

In the groups supplemented with CPLE, blood lead levels were substantially higher than in the NC group, showing values comparable to those in the PC group, with measurements ranging from 29.74 ± 0.65 μg/dL in the LD group to 21.24 ± 1.20 μg/dL in the VHD group. The VHD group exhibited a statistically significant decrease relative to the PC group (*p* < 0.01).

Liver Pb levels demonstrated a graded pattern across the different dosages. All CPLE supplemented groups demonstrated a statistically significant reduction in hepatic lead concentrations compared to the PC group (*p* < 0.01), with mean values decreasing from 1.83 ± 0.03 mg/kg in LD to 1.49 ± 0.06 mg/kg in HD. VHD showed a smaller decrease (1.20 ± 0.02 mg/kg; *p* < 0.01).

Kidney lead concentrations were significantly reduced in the MD, HD, and VHD groups in comparison to the PC group (*p* < 0.01). Values ranged from 3.01 ± 0.19 mg/kg in LD to 1.61 ± 0.07 mg/kg in VHD, with LD remaining comparable to PC.

Bone Pb levels were markedly elevated in all Pb-exposed groups relative to the negative control group. All groups supplemented with CPLE, except for VHD, demonstrated significantly lower bone Pb burdens relative to the PC group (*p* < 0.01). The HD and MD groups demonstrated the lowest values, recorded at 20.46 ± 0.27 mg/kg and 18.70 ± 0.56 mg/kg, respectively. The data reveal notable differences in Pb accumulation across tissues, with multiple CPLE-treated groups showing substantially lower Pb levels relative to the positive control.

**FIGURE 2:**
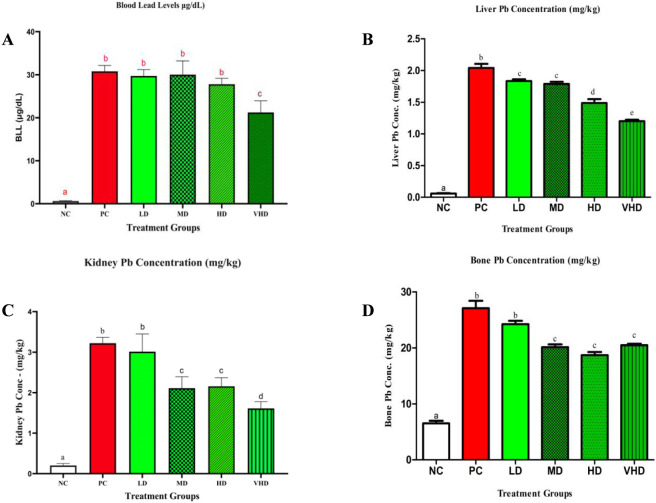
Pb bioaccumulation in blood (μg/dL) **(A)** liver (mg/kg) **(B)** kidney (mg/kg) **(C)** and bone (mg/kg) **(D)**. (Graphs: mean ± SEM, n = 5, a–e represents *p* < 0.05). Bars with different superscript letters differ significantly at *p* < 0.05 (ANOVA followed by *post hoc* test), shared letter symbols among groups indicate no significant difference and different letter symbols among groups denote significant difference. Treatment markedly reduced Pb burden in all examined tissues compared to the positive control, with the highest dose showing the most pronounced clearing effect.

Figure 2 graphically presents the effects of various treatments on intracellular Pb bioaccumulation. Overall, Pb accumulation in the tissues of exposed groups showed clear dose-dependent trend. In rats, exposure to PbAc resulted in a significant increase in Pb concentrations in the blood, liver, kidney, and bone compared with the normal control group (**NC**) (*p* < 0.001; *p* < 0.05). Among these tissues, Pb levels were highest in bone, followed by the kidney, liver, and blood. Supplementation with CPLE at 400 mg/kg BW corresponded with reduced Pb levels in blood, liver, and kidneys. Lower but significant reduced Pb levels were also observed with CPLE supplementation at doses of 50–200 mg/kg BW in the liver, 100–200 mg/kg BW in the kidney, and 100–300 mg/kg BW in bone. Compared with the PbAc-treated group (PC), CPLE supplementation corresponded with a more pronounced Pb levels in hepatic and renal tissues. However, no significant reduced Pb levels were observed in the blood and bone at lower CPLE doses (50–200 mg/kg BW and 50 mg/kg BW, respectively).

#### 3.4.1 Quality assurance data for AAS

To ensure reliability and accuracy of the data obtained from the AAS analysis of Pb, standards for each tissue batch were analyzed. Certified Reference Materials (CRMs) for soft tissues, hard tissues, and blood were analyzed concurrently with the experimental samples to verify analytical precision and accuracy.

Additionally, all Supplementary Material–including, distilled water, ultra-pure (double-distilled) water, rat feed (commercial pellets), and CPLs–were tested for Pb content to ensure the absence of contamination. The results of the standards, Instrument Detection Limit (IDL), Instrument Quantification Limit (IQL), recovery rates, and other quality control parameters were summarized Supplementary Table S8.

According to Supplementary Tables S8 and S9, and Supplementary Figure S6, recovery assessments using three certified reference materials demonstrated consistent analytical performance across different matrices. Pb concentrations measured in Seronorm (347.07 μg/L), DOLT-5 (0.191 μg/g), and Bone Ash (7.33 μg/g) resulted in recovery rates of 117.65%, 117.90%, and 80.82%, respectively, when compared to their certified values. All materials were subjected to duplicate analysis to ensure the reliability of measurements.

Supplementary Table S10 – (IDL and IQL values for AAS Pb analysis) above shows the obtained IDLs and their associated IQLs values as (0.0003/0.00100) mg/L for blood, 0.00128/0.00427 mg/L for liver, 0.00006/0.00020 mg/L for kidney, and (0.00016/0.00053) mg/L for bone.

Table 3 – (Pb levels in study materials–DW, DDW, feed, and *Carica papaya* leaves) above shows Pb concentrations in all study materials were significantly below the regulation limit of 10 ppm established by the WHO and FDA. The rat meal displayed a negligible lead concentration of 0.00008 ± 0.05 ppm, whereas Carica papaya leaf powder showed a slightly elevated but still minimal lead level of 0.00223 ± 0.87 ppm. Both DDW and DW samples exhibited undetectable levels of Pb.

**TABLE 3:** Pb levels in study materials (DW, DDW, feed, and *Carica papaya* leaves).

Sample type	Pb concentration (ppm)	WHO (ppm)	USA FDA (ppm)
Rat feed (pellets)	0.00008 ± 0.05	10.00	10.00
CPLP	0.00223 ± 0.87		
DDW	0		
DW	0		

Table 3 shows Pb concentrations in study materials [rat feed, CPLP (*Carica papaya* leaf powder), DDW, DW] in parts per million (ppm). The values were presented as mean ± standard deviation. Pb levels in all the analyzed samples were compared with WHO and FDA standard limits (10 ppm) (Zamir et al., 2015).

### 3.5 Assessment of lipid peroxidation through (MDA) assay in liver tissue

The concentrations of hepatic MDA in all the six groups are shown in Supplementary Table S12. The PC group exhibited a significant increase in MDA levels (38.49 ± 0.77 nmol/g) relative to the NC group (24.09 ± 0.55 nmol/g; *p* < 0.01). All groups supplemented with CPLE showed reduced MDA levels compared to the PC group, with the extent of reduction differing among the groups. LD (31.29 ± 1.11 nmol/g) and MD (28.81 ± 1.29 nmol/g) were substantially elevated relative to NC (*p* < 0.01) yet showed marked reductions in comparison to PC (*p* < 0.01). HD (24.19 ± 0.61 nmol/g) and VHD (22.47 ± 1.27 nmol/g) showed the lowest values among the treated groups, considerably lower than PC (*p* < 0.01). MDA concentrations demonstrated notable group-specific differences in response to Pb exposure and CPLE administration.

**FIGURE 3:**
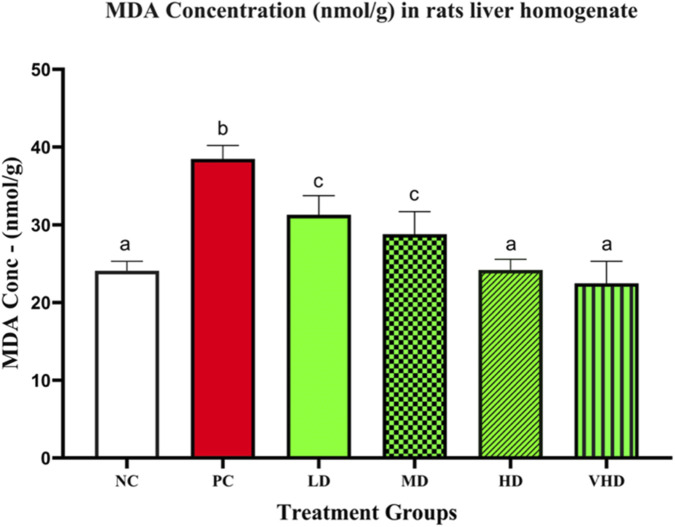
MDA levels in liver homogenate, in the absence of PbAc (NC), presence of PbAc only (PC) and CPLE supplementation (CPLE + PbAc) in rats. Values were expressed as mean ± SE, for five animals in each group, a–c represents p < 0.05. Shared letter symbols among groups indicated no significant difference and different letter symbols among groups denoted significant difference.

Figure 3 provide the tabular presentations of the MDA concentration levels in the analyzed liver homogenates after Pb exposure and CPLE supplementation in the experimental animals. The protective effect of CPLE against lipid peroxidation in the liver was assessed by measuring MDA levels in all experimental animals, as illustrated in Figure 3. Rats in the toxic control group (PC) demonstrated a substantial elevation in hepatic MDA levels relative to the untreated normal control (NC) group (*p* < 0.0001). Conversely, CPLE supplementation resulted in a notable decrease in MDA concentrations in all treatment groups relative to the toxic control, with *p*-values of 0.003 (LD), <0.0001 (MD), <0.0001 (HD), and <0.0001 (VHD).

## 4 Discussion

Pb is a widespread environmental and industrial contaminant that exerts toxic effects in diverse biological systems. While the exact Pb toxicity mechanism remains unclear, overwhelming evidence shows that Pb exposure leads to an excessive production of reactive oxygen and ntrogen species and depletes endogenous antioxidant defenses. The redox imbalance results in lipid peroxidation, protein and DNA damage, compromising cellular and tissue integrity (Dewanjee et al., 2013). Pb futher substitutes redox-active metals (e.g., Fe^2+^, Ca^2+^, Zn^2+^) in antioxidant enzymes including SOD, CAT, and GPx, disrupting mitochondrial electron transport, and altering nitric oxide synthase activity, which dysregulates NO signalling and promotes peroxynitrite (ONOO^−^) formation (Chandimali et al., 2025).

CPLE which contains phenolics and flavonoids could provide a viable, low-cost alternative source of exogenous naturally occurring antioxidants and chelators that could mitigate Pb-induced oxidative/nitrosative stress and ultimately Pb poisoning. This study investigated the effects of CPLE supplementation against Pb accumulation in tissues and its potential to reduce Pb-induced toxicity in rats.

The phytochemical analysis of CPLE confirmed the presence of phenolics and flavonoids, quantified using gallic acid and quercetin standards, respectively. These findings are consistent with previous reports on *C. papaya* leaf extracts (Arslan et al., 2022; Babalola et al., 2024; Kong et al., 2021; Thuppil and Tannir, 2013). The mean concentrations obtained from the assays were 177.86 ± 0.75 mg QEC/L and 741.73 ± 16.97 mg GAEC/L. Since the stock extract was prepared at a concentration of 1 mg/L, these assay values are directly equivalent to 177.86 mg QE/g and 741.73 mg GAE/g of extract. This indicates that the ethanolic extract of *C. papaya* leaves is a rich source of both flavonoids and phenolics.

Body weight is widely used as a broad measure of systemic toxicity in experimental toxicology; nevertheless, it is an integrative endpoint influenced by metabolic, endocrine, inflammatory, and behavioral processes rather than a direct proxy of cellular injury or recovery. Accordingly, changes in body weight observed in the present study must be interpreted in conjunction with biochemical and oxidative stress measures, rather than as independent evidence of toxicity attenuation.

During the exposure period, slight but heterogeneous changes in body weight were observed across experimental groups. A general reduction in body weight occurred between weeks 1 and 2 in the toxic control (PC) group and in all PbAc + CPLE–treated groups (LD, MD, HD, VHD), whereas no significant changes were detected in the normal control (NC) group. This early decline is consistent with Pb-induced metabolic disturbance and growth retardation reported in previous studies (Kang et al., 2004; Kataba et al., 2021). Mechanistically, Pb exposure is known to induce oxidative stress through excessive generation of ROS, leading to lipid peroxidation, protein oxidation, and mitochondrial dysfunction in metabolically active tissues such as the liver and skeletal muscle (Flora et al., 2012; Patrick, 2006). This is supported by elevated MDA levels and concomitant reductions in antioxidant defenses SOD, CAT, and GSH, indicating impaired redox homeostasis during the early exposure phase.

The sustained reduction in body weight observed in the PC, LD, and HD groups through week 3 suggests that Pb-induced metabolic impairment persists in the absence of adequate protective regulation. The MD group experienced significant weight reductions between weeks 1 and 2 (*p* < 0.0076) and the HD group between weeks 1 and 3 (*p* < 0.026), indicating non-linear, dose-dependent physiological responses to combined Pb and phytochemical exposure. Pb binds sulfhydryl (–SH) groups and affects metal homeostasis by displacing divalent cations such as Ca^2+^, Zn^2+^, and Fe^2+^. This inhibits enzymes involved in heme production, energy metabolism, lipid synthesis, and protein turnover (Gurer and Ercal, 2000). Inhibiting the Zn-dependent enzyme ALAD leads to metabolic inefficiency and decreased weight gain during persistent Pb exposure (Flora et al., 2012). Pb-induced Zn deficit worsens appetite suppression, intestinal barrier dysfunction, and gut microbiota disruption, limiting nutritional absorption and energy harvest (Kataba et al., 2021; Ninh et al., 1996). These pathways could explain the observed weight loss, despite modest biochemical improvements in some CPLE-treated groups.

Importantly, the absence of uniform weight recovery in CPLE-treated groups could not negate its protective effects at the molecular level. CPLE supplementation resulted in reduced MDA and Pb levels in metabolically active tissue such as the liver tissue which could be partially correlated to reduced lipid peroxidation/oxidative stress and Pb burden. The dissociation between biochemical recovery and body weight normalization could indicate that improvement in cellular redox status and organ function may precede or occur independently of gross changes in body mass. This phenomenon has been reported in other studies where antioxidant or chelating interventions improved biochemical and histopathological endpoints without fully restoring body weight (Flora et al., 2012; Gurer and Ercal, 2000).

Notably, a significant weight gain (*p*<0.035) was observed in the VHD group receiving the highest CPLE dose (400 mg/kg BW). This finding suggests a threshold-dependent effect of CPLE, wherein higher concentrations of flavonoids and phenolic compounds may more effectively counteract Pb-induced oxidative stress and tissue Pb accumulation (Heim et al., 2002; Sharma et al., 2020). Enhanced antioxidant capacity at this dose may facilitate partial restoration of essential metal balance, including Zn^2+^, thereby improving appetite regulation, gut function, and overall metabolic efficiency. The concomitant biochemical improvements observed in this group support the interpretation that weight gain at higher CPLE doses reflects functional recovery rather than nonspecific anabolic effects.

Additionally, phytochemicals are known to exert metabolic actions, including modulation of lipid metabolism, mitochondrial efficiency, and gut microbiota composition, which may influence body weight irrespective of toxic burden (Williamson and Clifford, 2017). Therefore, weight fluctuations in certain CPLE-treated groups likely reflect adaptive metabolic responses during detoxification and tissue repair rather than persistent or exacerbated toxicity. At higher doses, however, phytochemicals such as alkaloids, phenolics, and flavonoids present in *C. papaya* leaves may exert mild metabolic or gastrointestinal effects that attenuate further weight gain despite continued supplementation. Thus, the absence of a linear dose–response relationship for body weight gain may reflect a threshold beyond which higher doses do not confer additional growth benefits. This interpretation is consistent with reports that higher doses of some plant extracts may shift from nutritive to regulatory or mildly stress-inducing effects (Calabrese and Baldwin, 2003).

In this study, Pb exposure caused significant Pb accumulation in tissues of the experimental animals. Although the lead acetate dose used does not directly reflect typical human or animal environmental exposure levels, it was selected to model cumulative lead toxicity under controlled sub-chronic conditions, enabling reproducible tissue accumulation and toxicological responses (Leão et al., 2020). This approach permits mechanistic evaluation of lead-induced effects while acknowledging the limitations of extrapolating administered doses to real-world environmental exposure scenarios (Leão et al., 2020).

Oral gavage was used to ensure controlled systemic exposure, with Pb entering the gastrointestinal tract, followed by absorption into the bloodstream and subsequent deposition in hard and soft tissues, consistent with established lead toxicokinetics (Flora et al., 2012).

In this present study CPLE supplementation was proposed to mitigate Pb tissue uptake and ameliorate Pb-induced toxicity. Many authors have reported that plant flavonoids and phenolics could reduce the accumulation of heavy metals including Pb in tissues of biological systems (Arslan et al., 2022; Dewanjee et al., 2013; Francis et al., 2020; Guo et al., 2024; Heim et al., 2002; Liu et al., 2013; Liu et al., 2010; Okon et al., 2023). In this study, flavonoids and phenolics enriched CPLE supplementation corresponded with decreased Pb levels in blood, liver, kidney and partly in bone, which provide preliminary data for its potential of having a protective effect against Pb–induced toxicity. Notably, significant reductions in Pb levels (*p* < 0.05) were observed at 400 mg/kg BW CPLE supplementation in the blood and at doses ranging from 100 to 400 mg/kg BW in bone, suggesting that higher doses of CPLE may enhance Pb clearance in these tissues. In contrast, a clear dose-dependant decrease in Pb accumulation was observed in liver and kidney. The observed effects may be attributed to the metal-chelating properties of flavonoids and phenolic compounds present in CPLE, which likely facilitated Pb mobilization and elimination from tissues of exposed animals (Dewanjee et al., 2013; Francis et al., 2020; Heim et al., 2002; Liu et al., 2013; Liu et al., 2010; Okon et al., 2023; Sharma et al., 2020). Our findings, however, also demonstrated a non-monotonic dose-response relationship for CPLE in kidney and bone tissues, with higher dosages not providing proportionally more protection. Specifically, the VHD (400 mg/kg BW) in bone and the HD (200 mg/kg BW) in kidney showed lack of superior protection when compared to their immediately preceding lower doses. Such non-linear responses are typical of hormetic dose-response relationships, which are well documented for phytochemicals and antioxidants. They are frequently attributed to saturation of protective pathways, activation of counter-regulatory mechanisms, or altered cellular redox signaling at higher concentrations. Similar non-monotonic biological responses to bioactive plant chemicals across tissues have been extensively characterized and mechanistically defined under the hormesis paradigm (Calabrese and Baldwin, 2003). Bone, unlike soft tissues, showed no reduction in Pb accumulation at the VHD (400 mg/kg BW), with Pb levels not significantly different from those in the MD and HD groups. This is consistent with bone acting as a long-term Pb reservoir, retaining up to ∼90% of the total body burden with very slow turnover and strong incorporation of Pb into the calcified hydroxyapatite matrix, allowing persistence for decades (Generalova et al., 2025). Human and animal chelation studies demonstrate that chelatable Pb primarily reflects blood and soft-tissue pools, while skeletal Pb is poorly mobilized (Tell et al., 1992; Zhang et al., 2019). Together, these findings indicate that beyond a threshold, increasing CPLE dose is unlikely to enhance Pb chelation from mineralized tissue, and higher doses may preferentially reduce circulating or soft-tissue Pb rather than Pb sequestered in bone, explaining the lack of added benefit of the VHD over MD and HD (Generalova et al., 2025; Tell et al., 1992; Zhang et al., 2019).

The certified reference materials Seronorm (117.65%) and DOLT-5 (117.90%) had slightly higher recovery rates, indicating a slight positive bias that might be due to a negligible matrix-induced enhancement effect or analytical uncertainty. However, these recoveries do not undermine the validity of the analytical data and are within widely accepted acceptable limits for biological metal analysis (Thompson et al., 2002). In contrast, the lower recovery for Bone Ash (80.82%) underscores challenges in digesting highly mineralized materials, while confirming the thoroughness of digestion and analytical accuracy (Gende and Schmeling, 2022; Tehrani et al., 2020). The digestion process was validated by these recovery patterns, accounting for matrix effects in quantitative metal analyses (Panuwet et al., 2016).

The method’s sensitivity, demonstrated by matrix-specific IDL/IQL values; (0.0003/0.00100 mg/L for blood, 0.00006/0.00020 mg/L for kidney, and 0.00016/0.00053 mg/L for bone), guarantees accurate detection of lead at low endogenous concentrations appropriate for biomonitoring environmental exposures (Belter et al., 2014; Kummrow et al., 2008; Rodríguez-Saldaña et al., 2021). Analysis of potential external contamination sources, such as feed, water, and *Carica papaya* leaves, indicated lead levels significantly below international regulatory thresholds (10 ppm limit established by WHO and FDA), thereby confirming that husbandry practices did not introduce measurable exogenous lead (Tadić et al., 2025; Vorkamp et al., 2021; Zamir et al., 2015). The negligible lead concentrations in rat meal (0.00008 ± 0.05 ppm) and leaf powder (0.00223 ± 0.87 ppm), coupled with the lack of detectable lead in distilled and de-ionized water, substantiate the reliability of biological test results as indicators of experimental exposure rather than environmental contamination (Tadić et al., 2025; Vorkamp et al., 2021). This result corroborates the analytical methodology for lead biomonitoring, exhibiting reliable quality control and sensitivity across diverse biological matrices.

Lipid peroxidation is the oxidative degradation of cell membrane lipids by reactive oxygen species resulting in cellular and tissue damage. MDA is a specific product of lipid peroxidation, which is usually used as a biomaker of lipid peroxidation as well as oxidative stress. MDA reacts with thiobarbituric acid to produce a pink coloured complex which is detectable spectrophotometrically, hence used quantitavely to measure lipid peroxidation and oxidative stress. Several aldehydes and certain sugars, including MDA, can react with thiobarbituric acid (TBA) and are therefore collectively called Thiobarbituric Acid Reacting substances (TBARs). Among these, MDA is the predominant contributor to the formation of the coloured MDA-TBA complex. Elevated MDA levels are indicative of increased lipid peroxidation (Dewanjee et al., 2013; Liu et al., 2013). Pb is also known to induce heavy metal-associated oxidative stress, primarily through the peroxidation of cell membrane lipids. In the present study, Pb exposure led to a marked increase in hepatic MDA levels in the experimental animals. MDA concentrations were determined from liver homogenates to assess the extent of lipid peroxidation. Supplementation with CPLE corresponded with significant reduced MDA levels (*p* < 0.05) in a dose-dependant manner. This could suggest an ameliorative effect of CPLE, likely attributable to the potent free radical-scavenging activity of flavonoids and phenolic compounds present it. These findings are consistent with previous reports demonstrating the antioxidant and hepatoprotective properties of plant-derived polyphenols (Apak et al., 2016; Dewanjee et al., 2013; Flora and Pachauri, 2010).

## 5 Conclusion

In conclusion, this study provides preliminary evidence supporting the potential of CPLE to mitigate Pb-induced toxicity in rats. Phytochemical screening confirmed the presence of flavonoids and phenolic compounds in CPLE, providing a biochemical basis for its observed biological effects. Pb exposure altered growth parameters, and although CPLE supplementation was associated with variable body-weight responses across treatment groups, these changes were not consistent and should be interpreted cautiously as supportive rather than definitive indicators of toxicity attenuation.

Pb concentrations were quantified in blood, liver, kidney, and bone tissues, with CPLE supplementation particularly at higher doses (>100 mg/kg BW) corresponding to reduced Pb accumulation across these tissues. Consistently, decreased hepatic MDA levels in CPLE-treated groups suggest attenuation of Pb-induced lipid peroxidation and hepatic oxidative stress.

While these findings are encouraging, they remain preliminary. Further studies are required to clarify the physiological relevance of growth-related changes and to elucidate underlying protective mechanisms, including antioxidant gene expression (CAT, SOD, and GPx), targeted metal–ligand computational analyses, and comprehensive clinical, safety, and toxicity evaluations. Nonetheless, given the established nutritional value and documented safety of *Carica papaya* fruit and leaf consumption (Francis et al., 2020; Halim et al., 2011; Rani et al., 2023; Taychaworaditsakul et al., 2024), dietary incorporation of CPLE may offer a promising dual benefit of nutritional enrichment and mitigation of Pb-induced oxidative toxicity.

Overall, these results position CPLE as a candidate for further investigation into accessible, diet-based strategies for heavy-metal toxicity mitigation, pending confirmation through rigorous mechanistic and translational studies.

### 5.1 Recommendations

Further studies should incorporate advanced more specific analytical platforms, such as HPLC or LC-MS/MS, for precise phytochemical profiling and quantification of oxidative stress markers.

Antioxidant gene expression studies and enzymatic rate assays such as SOD, CAT, GPx, and Nrf2 signaling will be useful to demonstrate molecular pathways. Targeted metal-ligand computational investigations would aid in defining the plausibility and specificity of interactions between Pb and the bioactive components of CPLE, thereby strengthening the mechanistic rationale for its potential use in mitigating Pb-induced toxicity.

The inclusion of comprehensive renal and hepatic function testing, as well histopathology studies in future studies would provide more evidence for systemic recovery.

Future research should prioritize leaf collection during the late dry season (August–September), when stress-responsive phenylpropanoid flux is maximized (Toscano et al., 2019). At this stage, heightened irradiance, cumulative ultra-violet (UV) exposure, and progressive water deficit act as strong abiotic signals that stimulate secondary metabolism, thereby enhancing phytochemical accumulation and bioactivity (Toscano et al., 2019). Such optimization would not only increase the yield of flavonoids and phenolics in ethanolic extracts but also strengthen the translational potential of *Carica papaya* as a phytotherapeutic resource.

Lastly, inclusion of a group receiving CPLE alone without Pb exposure is recommended for future studies to enable assessment of the extract’s intrinsic effects, safety, or metabolic impact independent of lead toxicity.

### 5.2 Limitations

Phytochemical characterization was limited to spectrophotometric, assay-based quantification of total flavonoid and total phenolic contents using a 96-well microplate reader. While informative, these approaches lack the precision and compound-specific resolution afforded by advanced analytical techniques such as HPLC or LC-MS/MS, which would enable accurate identification and quantification of individual phytoconstituents.

Similarly, oxidative stress assessment was restricted to the measurement of MDA levels in liver homogenates. Although this provided some insight into CPLE’s potential to attenuate Pb-induced lipid peroxidation, it represents only a single aspect of oxidative stress status. The inclusion of complementary antioxidant and redox markers such as SOD, CAT, GPx, or total antioxidant capacity would have strengthened conclusions regarding oxidative stress modulation and antioxidant defense mechanisms.

Additionally, the *Carica papaya* leaves used in this study were harvested in June, corresponding to the early dry season in Lusaka, Zambia. During this period, plants typically experience moderate light intensity and minimal water stress, conditions under which activation of the phenylpropanoid pathway may be limited. This seasonal context could have constrained the accumulation of flavonoids and phenolic compounds in the ethanolic extracts, as previously reported (Toscano et al., 2019).

Finally, financial constraints precluded the inclusion of antioxidant gene expression analyses, histopathological studies, enzymatic activity assays, and comprehensive renal and hepatic function tests. The absence of these endpoints limits the extent to which conclusions can be drawn regarding systemic physiological restoration and mechanistic pathways underlying the observed protective effects.

## Data Availability

The original contributions presented in the study are included in the article/Supplementary Material, further inquiries can be directed to the corresponding author.
