# Supplementary material for: Protective effect of Carica papaya ethanolic leaf extract against lead-induced toxicity in Wistar rats
Source: Front Toxicol. 2026 Apr 10;8:1762172. doi: 10.3389/ftox.2026.1762172 (PMC13105474; doi:10.3389/ftox.2026.1762172)
Supplement: Supplementary file 1 [file DataSheet1.docx]

Supplementary Material

**CONTENTS:**

**1. MATERIALS AND METHOD**

**Tables – 1**

**Table S1:** Operating conditions for SpeedWave MWS-2 microwave digestion.

**Table S2:** Operating conditions setup for AAS

**Table S3:** MDA reagents and sample assay preparation guide.

**Figures – 1**

Figure S1: Workflow for Step-by-step Extraction Process of CPLE.

Figure S2: Workflow for tissue processing for Pb quantification

Figure S3: Workflow for liver tissue processing for MDA analysis

**2. RESULTS**

**Tables – 2**

**Table S4.** Absorbance values for gallic acid standard (std) curve.

**Table S5:** Absorbance values for quercetin std curve.

**Table S6:** Effect of PbAc and CPLE on animal weight change

**Table S7:** Effect of Pb bioaccumulation in blood, liver, kidney and bone

**Table S8:** Standard curve data for Pb analysis in tissues.

**Table S9:** Certified reference materials recovery rate results

**Table S10:** IDL and IQL values for AAS Pb analysis

**Table S11:** Assessment of MDA in liver tissue

**Table S12:** Supplementary Results Data

**Figures – 2**

**Figure S4:** Absorbance against concentration of gallic acid standard solution for estimation of total phenolic content (expressed as gallic equivalent concentration (GEC) in mg/l) in CPLE. The determined standard curve had an R^2^ value of 0.9940.

**Figure S5:** Absorbance against concentration of quercetin standard solution for determination of total flavonoid content (expressed as quercetin equivalent concentration (QEC) in mg/l) in the CPLE. The determined standard curve had an R^2^ value of 0.9989.

**Figure S6:** Calibration curves used for Pb quantification across different biological matrices. Standard calibration plots were generated for blood, liver, kidney, and bone samples using AAS; Z-2010 series. Each curve displays the corresponding linear regression equation and coefficient of determination (R²), indicating good linearity across the tested concentration range.

1. **MATERIALS AND METHOD**

**Tables – 1**

| Soft tissues (Liver and Kidney) | | Hard tissue (Bone) | | Blood | | Vegetation (CPL) | |
| --- | --- | --- | --- | --- | --- | --- | --- |
| Temp. (ºC) | Time (min.) | Temp. (ºC) | Time (min.) | Temp. (ºC) | Time (min.) | Temp. (ºC) | Time (min.) |
| 160 | 5 | 150 | 5 | 160 | 5 | 150 | 5 |
| 190 | 20 | 175 | 5 | 190 | 10 | 175 | 5 |
| 200 | 20 | 200 | 20 | 75 | 10 | 200 | 20 |
| 100 | 5 | 180 | 10 | 0 | 0 | 180 | 10 |

**Table S1.** Operating conditions for SpeedWave MWS-2 microwave digestion.

**Table S1** shows the operating conditions for microwave digestion for blood, liver, kidney, bone and vegetation

**Table S2.** Operating conditions setup for AAS

| Parameter | Value |
| --- | --- |
| Signal Mode | BKG Correct. |
| Calculation Mode | Peak Height |
| Wavelength (nm) | 283.3 |
| Wavelength Setting | Automatic |
| Slit Width (nm) | 1.3 |
| Time Constant (s) | 0.1 |
| Lamp Current (mA) | 7.5 |
| PMT Voltage (V) | 30 s |

**Table S2**. Shows the AAS operating instrument conditions setup used for the analyses of the study samples.

| Reagent (μL) | Assay tube | Blank tube |
| --- | --- | --- |
| MDA Detection Working Solution | 300 | 300 |
| Distilled Water |  | 100 |
| Sample | 100 |  |
| Reagent 3 (Stabilizer)* | 100 | 100 |

**Table S3**. MDA reagents and sample assay preparation guide.

Table S3. Shows a list of reagent volumes required to perform an MDA assay including the blank setup. * Reagent 3 contains sodium acetate buffer, sodium chloride, sodium azide and EDTA as stabilizers.

**Figures – 1**

Figure S1. Workflow for the step-by-step extraction process of CPLE.

Figure S2. Workflow for tissue processing for Pb quantification

**Figure S3**. Workflow for liver tissue processing for MDA analysis

1. **RESULTS**

**Tables – 2**

| Sample ID | Blank | Std 1 | Std 2 | Std 2 | Std 3 | Std 4 | Std 5 | Std 6 |
| --- | --- | --- | --- | --- | --- | --- | --- | --- |
| Conc. (ppm or mg/l) | 0 | 5 | 100 | 200 | 400 | 1000 | 1500 | 2000 |
| Absorbance (765 nm) | 0.0000 | 0.0037 | 0.0147 | 0.0877 | 0.2457 | 0.5200 | 0.7360 | 1.0897 |

**Table S4.** Absorbance values for gallic acid standard (std) curve.

Table S4. Concentrations and corresponding absorbance values of gallic acid standards measured at 765 nm, used to construct the calibration curve for TPC quantification.

| Sample ID | Blank | Std 1 | Std 2 | Std 2 | Std 3 | Std 4 | Std 5 |
| --- | --- | --- | --- | --- | --- | --- | --- |
| Conc. (ppm or mg/l) | 0.000 | 5 | 10 | 20 | 40 | 80 | 100 |
| Absorbance (510 nm) | 0.0000 | 0.0093 | 0.0206 | 0.0313 | 0.0726 | 0.1473 | 0.1853 |

**Table S5.** Absorbance values for quercetin std curve.

Table S5. Concentrations and corresponding absorbance values of quercetin standards measured at 510 nm, used to construct the calibration curve for TFC quantification.

| **Treatment**  **Group** | **NC** | **PC** | **LD** | **MD** | **HD** | **VHD** |
| --- | --- | --- | --- | --- | --- | --- |
|  | **(Mean/SD)** | **(Mean/SD)** | **(Mean/SD)** | **(Mean/SD)** | **(Mean/SD)** | **(Mean/SD)** |
| **Week 1** | 195.1 + 22.77 | 195.6 + 30.9 | 177.8 + 24.92 | 214.3 + 36.17 | 212.5 + 21.10 | 210.7 + 29.26 |
| **Week 2** | 200.5 + 22.23 | 191.2 + 29.28 | 170.3 + 33.44 | 207.3 + 35.23 | 203.2 + 22.75 | 197.0 + 36.39 |
| **Week 3** | 197.0 + 24.67 | 188.2 + 29.29 | 162.5 + 26.41 | 210 + 36.34 | 200.8 + 20.96 | 198.8 + 29.43 |
| **Week 4** | 199.8 + 23.49 | 191.9 + 35.88 | 167.6 + 17.63 | 210.1 + 33.37 | 202.2 + 18.07 | 207.2 + 26.53 |
| **Week 5** | 194.1 + 46.11 | 195.2 + 34.09 | 172.4 + 16.92 | 207.2 + 28.15 | 201.2 + 18.23 | 206.1 + 33.07 |

**Table S6.** Effect of PbAc and CPLE on animal weight change

**Table S6** shows weekly weights (mean + SD) of experimental animals (rats) per exposure group NC - (50mg/Kg BW DDW)), PC (standard treatment - 50mg/Kg BW, PbAc only), LD (standard treatment + CPLE 50mg/Kg B.W), MD (standard treatment + CPLE 100mg/Kg B.W), HD (Standard Treatment + CPLE 200mg/Kg B.W), and VHD (Standard Treatment + CPLE 400mg/Kg B.W) taken in five weeks.

| Groups | Pb burden | | | |
| --- | --- | --- | --- | --- |
|  | **Blood (μg/dl)** | **Liver (mg/kg of wet weight)** | **Kidney (mg/kg of wet weight)** | **Bone (mg/kg of wet weight)** |
| NC | 0.62 + 0.06 | 0.06 + 0.01 | 0.20 + 0.02 | 6.53 + 0.44 |
| PC | 30.78 + 0.61# | 2.04 + 0.07# | 3.22 + 0.06# | 27.07 + 1.33# |
| LD | 29.74 + 0.65# | 1.83 + 0.03#* | 3.01 + 0.19# | 20.12 + 0.51#** |
| MD | 30.02 + 1.42# | 1.79 + 0.03#** | 2.11 + 0.13#** | 18.70 + 0.56#** |
| HD | 27.80 + 0.62# | 1.49 + 0.06#** | 2.16 + 0.09#** | 20.46 + 0.27#** |
| VHD | 21.24 + 1.20#** | 1.20 + 0.02#** | 1.61 + 0.07#** | 24.22 + 0.61# |

**Table S7.** Effect of Pb bioaccumulation in blood, liver, kidney and bone

Table S7 shows Values expressed as mean ± SE, for five animals in each group. # Values differ significantly from NC (normal control) (*p* < 0.01). * Values differ significantly from PC (positive control-Pb-acetate control) (*p* < 0.05). ** Values differ significantly from PC (positive control-Pb-acetate control) (*p* < 0.01).

| Concentration (ppb) | Absorbance (nm) | | | |
| --- | --- | --- | --- | --- |
|  | **Blood** | **Liver** | **Kidney** | **Bone** |
| 0 | 0.00 | 0.00 | 0.00 | 0.00 |
| 5 | 0.03 | 0.03 | 0.02 | 0.03 |
| 10 | 0.06 | 0.05 | 0.05 | 0.05 |
| 25 | 0.13 | 0.14 | 0.12 | 0.13 |
| 50 | 0.24 | 0.25 | 0.21 | 0.24 |

**Table S8.** Standard curve data for Pb analysis in tissues

Table S8 shows the concentrations and corresponding absorbance values obtained for blood, liver, kidney, and bone matrices, used to generate the respective standard calibration curves.

| Parameter | Observed Value | CRM Reference Value | Recovery  Rate  (%) |
| --- | --- | --- | --- |
| (CRM) Matrix |  |  |  |
| Seronorm (μg/L) - (SERO AS Stasjonsveien 44, NO-1396 Billingstad, Norway) | 347.07 | 295.00 | 117.65 |
| DOLT-5 (μg/g) - (Dogfish liver; National Research Council of Canada) | 0.191 | 0.162 | 117.90 |
| Bone Ash (μg/g) - (SRM 1400, Monsanto Co., St. Louis, MO, USA) | 7.33 | 9.07 | 80.82 |

**Table S9.** Certified reference materials recovery rate results

Table S9 shows three CRMs for blood (Seronorm), soft tissue (liver and kidney) -DOLT-5, and hard tissue (bone)-Bone Ash) and their recovery rates. The CRMs were prepared and analyzed in triplicate to ensure accuracy of results.

**Table S10.** IDL and IQL values for AAS Pb analysis

| Tissue | (IDL)  mg/l | (IQL)  mg/l |
| --- | --- | --- |
| Blood | 0.00030 | 0.00100 |
| Liver | 0.00128 | 0.00427 |
| Kidney | 0.00006 | 0.00020 |
| Bone | 0.00016 | 0.00053 |

Table S10 shows the Instrument Detection Limits (IDLs), and Instrument Quantification Limits (IQLs) determined by AAS -graphite mode per specific tissue analyzed. IQL was calculated as a 10/3 of IDL as described by Belter et al., (2014) .

|  | Groups | | | | | | |
| --- | --- | --- | --- | --- | --- | --- | --- |
|  | **NC** | **PC** | **LD** | **MD** | **HD** | **VHD** | |
| MDA (nmol/g) | 24.09 + 0.55 | 38.49 + 0.77# | 31.29 + 1.11#** | 28.81 + 1.29# | 24.19 + 0.61** | | 22.47 + 1.27** |

**Table S11.** Assessment of MDA in liver tissue

Table S12 shows MDA values expressed as mean ± SE, for six treatment groups; NC - (50mg/Kg BW DDW)), PC (standard treatment - 50mg/Kg BW, PbAc only), LD (standard treatment + CPLE 50mg/Kg B.W), MD (standard treatment + CPLE 100mg/Kg B.W), HD (Standard Treatment + CPLE 200mg/Kg B.W), and VHD (Standard Treatment + CPLE 400mg/Kg B.W) . # Values differ significantly from NC (normal control) (p < 0.01). * Values differ significantly from PC (positive control-Pb-acetate control) (p < 0.05). ** Values differ significantly from PC (positive control-Pb-acetate control) (p < 0.01).

**Figures – 2**

Figure S4. Gallic acid standard curve – Absorbance versus Concentration (mg/l or ppm).

Figure S5. Quercetin Standard Curve – Absorbance versus Concentration (mg/l or ppm).


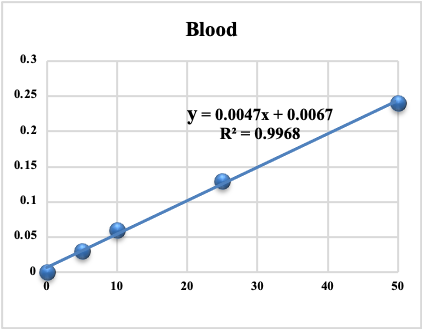

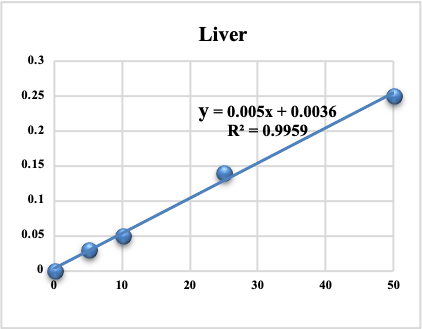

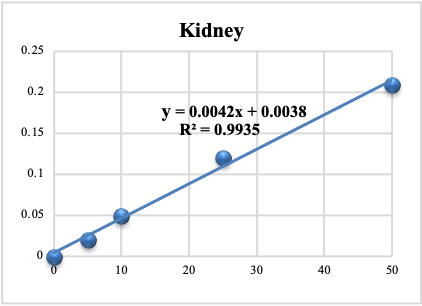

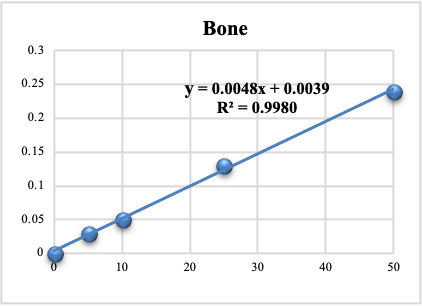


**Figure S6.** Calibration curves for Pb analysis in tissues

## Table S12. Supplementary Results Data

| Organ/Tissue | Comparison | Mean 1 | Mean 2 | Mean diff. | SE of diff. | 95.00% CI of diff. | Below threshold? | Summary | Adjusted *P* Value | t | DF |
| --- | --- | --- | --- | --- | --- | --- | --- | --- | --- | --- | --- |
|  |  |  |  |  |  |  |  |  |  |  |  |
| Blood | NC - PC | 0.62 | 30.78 | -30.16 | 0.612 | -33.32 to -27.00 | Yes | **** | <0.0001 | 49.28 | 4.065 |
| Blood | NC - LD | 0.62 | 29.74 | -29.12 | 0.6553 | -32.51 to -25.73 | Yes | **** | <0.0001 | 44.44 | 4.056 |
| Blood | NC - MD | 0.62 | 30.02 | -29.4 | 1.425 | -36.76 to -22.04 | Yes | *** | 0.0003 | 20.63 | 4.012 |
| Blood | NC - HD | 0.62 | 27.8 | -27.18 | 0.6216 | -30.39 to -23.97 | Yes | **** | <0.0001 | 43.73 | 4.063 |
| Blood | NC - VHD | 0.62 | 21.24 | -20.62 | 1.204 | -26.84 to -14.40 | Yes | *** | 0.0006 | 17.12 | 4.017 |
| Blood | PC - LD | 30.78 | 29.74 | 1.04 | 0.8933 | -2.436 to 4.516 | No | ns | 0.9597 | 1.164 | 7.962 |
| Blood | PC - MD | 30.78 | 30.02 | 0.76 | 1.549 | -6.382 to 7.902 | No | ns | >0.9999 | 0.4906 | 5.418 |
| Blood | PC - HD | 30.78 | 27.8 | 2.98 | 0.8689 | -0.4007 to 6.361 | No | ns | 0.0926 | 3.43 | 7.998 |
| Blood | PC - VHD | 30.78 | 21.24 | 9.54 | 1.349 | 3.774 to 15.31 | Yes | ** | 0.0042 | 7.073 | 5.927 |
| Blood | LD - MD | 29.74 | 30.02 | -0.28 | 1.567 | -6.978 to 6.418 | No | ns | >0.9999 | 0.1787 | 5.611 |
| Blood | LD - HD | 29.74 | 27.8 | 1.94 | 0.8999 | -1.561 to 5.441 | No | ns | 0.4739 | 2.156 | 7.978 |
| Blood | LD - VHD | 29.74 | 21.24 | 8.5 | 1.369 | 2.648 to 14.35 | Yes | ** | 0.0083 | 6.21 | 6.169 |
| Blood | MD - HD | 30.02 | 27.8 | 2.22 | 1.553 | -4.939 to 9.379 | No | ns | 0.8568 | 1.43 | 5.46 |
| Blood | MD - VHD | 30.02 | 21.24 | 8.78 | 1.864 | 1.527 to 16.03 | Yes | * | 0.0175 | 4.71 | 7.783 |
| Blood | HD - VHD | 27.8 | 21.24 | 6.56 | 1.353 | 0.7754 to 12.34 | Yes | * | 0.0282 | 4.848 | 5.98 |
| Liver | NC - PC | 0.0582 | 2.04 | -1.981 | 0.05969 | -2.166 to -1.797 | Yes | *** | <0.0001 | 46.95 | 24 |
| Liver | NC - LD | 0.0582 | 1.833 | -1.775 | 0.05969 | -1.959 to -1.590 | Yes | *** | <0.0001 | 42.05 | 24 |
| Liver | NC - MD | 0.0582 | 1.789 | -1.731 | 0.05969 | -1.915 to -1.546 | Yes | *** | <0.0001 | 41.01 | 24 |
| Liver | NC - HD | 0.0582 | 1.486 | -1.428 | 0.05969 | -1.613 to -1.243 | Yes | *** | <0.0001 | 33.84 | 24 |
| Liver | NC - VHD | 0.0582 | 1.2 | -1.142 | 0.05969 | -1.327 to -0.9576 | Yes | *** | <0.0001 | 27.06 | 24 |
| Liver | PC - LD | 2.04 | 1.833 | 0.2066 | 0.05969 | 0.02204 to 0.3912 | Yes | * | 0.02 | 4.895 | 24 |
| Liver | PC - MD | 2.04 | 1.789 | 0.2508 | 0.05969 | 0.06624 to 0.4354 | Yes | ** | 0.0038 | 5.943 | 24 |
| Liver | PC - HD | 2.04 | 1.486 | 0.5534 | 0.05969 | 0.3688 to 0.7380 | Yes | *** | <0.0001 | 13.11 | 24 |
| Liver | PC - VHD | 2.04 | 1.2 | 0.8392 | 0.05969 | 0.6546 to 1.024 | Yes | *** | <0.0001 | 19.88 | 24 |
| Liver | LD - MD | 1.833 | 1.789 | 0.0442 | 0.05969 | -0.1404 to 0.2288 | No | ns | 0.9746 | 1.047 | 24 |
| Liver | LD - HD | 1.833 | 1.486 | 0.3468 | 0.05969 | 0.1622 to 0.5314 | Yes | *** | <0.0001 | 8.217 | 24 |
| Liver | LD - VHD | 1.833 | 1.2 | 0.6326 | 0.05969 | 0.4480 to 0.8172 | Yes | *** | 0.0004 | 14.99 | 24 |
| Liver | MD - HD | 1.789 | 1.486 | 0.3026 | 0.05969 | 0.1180 to 0.4872 | Yes | *** | 0.0009 | 7.17 | 24 |
| Liver | MD - VHD | 1.789 | 1.2 | 0.5884 | 0.05969 | 0.4038 to 0.7730 | Yes | *** | <0.0001 | 13.94 | 24 |
| Liver | HD - VHD | 1.486 | 1.2 | 0.2858 | 0.05969 | 0.1012 to 0.4704 | Yes | *** | <0.0001 | 6.772 | 24 |
| Kidney | NC - PC | 0.202 | 3.22 | -3.018 | 0.1556 | -3.499 to -2.537 | Yes | **** | <0.0001 | 27.43 | 24 |
| Kidney | NC - LD | 0.202 | 3.014 | -2.812 | 0.1556 | -3.293 to -2.331 | Yes | **** | <0.0001 | 25.56 | 24 |
| Kidney | NC - MD | 0.202 | 2.112 | -1.91 | 0.1556 | -2.391 to -1.429 | Yes | **** | <0.0001 | 17.36 | 24 |
| Kidney | NC - HD | 0.202 | 2.158 | -1.956 | 0.1556 | -2.437 to -1.475 | Yes | **** | <0.0001 | 17.78 | 24 |
| Kidney | NC - VHD | 0.202 | 1.61 | -1.408 | 0.1556 | -1.889 to -0.9269 | Yes | **** | <0.0001 | 12.8 | 24 |
| Kidney | PC - LD | 3.22 | 3.014 | 0.206 | 0.1556 | -0.2751 to 0.6871 | No | ns | 0.7693 | 1.872 | 24 |
| Kidney | PC - MD | 3.22 | 2.112 | 1.108 | 0.1556 | 0.6269 to 1.589 | Yes | **** | <0.0001 | 10.07 | 24 |
| Kidney | PC - HD | 3.22 | 2.158 | 1.062 | 0.1556 | 0.5809 to 1.543 | Yes | **** | <0.0001 | 9.652 | 24 |
| Kidney | PC - VHD | 3.22 | 1.61 | 1.61 | 0.1556 | 1.129 to 2.091 | Yes | **** | <0.0001 | 14.63 | 24 |
| Kidney | LD - MD | 3.014 | 2.112 | 0.902 | 0.1556 | 0.4209 to 1.383 | Yes | **** | <0.0001 | 8.198 | 24 |
| Kidney | LD - HD | 3.014 | 2.158 | 0.856 | 0.1556 | 0.3749 to 1.337 | Yes | *** | 0.0002 | 7.78 | 24 |
| Kidney | LD - VHD | 3.014 | 1.61 | 1.404 | 0.1556 | 0.9229 to 1.885 | Yes | **** | <0.0001 | 12.76 | 24 |
| Kidney | MD - HD | 2.112 | 2.158 | -0.046 | 0.1556 | -0.5271 to 0.4351 | No | ns | 0.9997 | 0.4181 | 24 |
| Kidney | MD - VHD | 2.112 | 1.61 | 0.502 | 0.1556 | 0.02088 to 0.9831 | Yes | * | 0.0373 | 4.562 | 24 |
| Kidney | HD - VHD | 2.158 | 1.61 | 0.548 | 0.1556 | 0.06688 to 1.029 | Yes | * | 0.0191 | 4.981 | 24 |
| Bone | NC - PC | 6.528 | 27.07 | -20.55 | 0.9983 | -23.63 to -17.46 | Yes | **** | <0.0001 | 29.11 | 24 |
| Bone | NC - LD | 6.528 | 20.12 | -13.59 | 0.9983 | -16.67 to -10.50 | Yes | **** | <0.0001 | 19.25 | 24 |
| Bone | NC - MD | 6.528 | 18.7 | -12.17 | 0.9983 | -15.26 to -9.083 | Yes | **** | <0.0001 | 17.24 | 24 |
| Bone | NC - HD | 6.528 | 20.46 | -13.93 | 0.9983 | -17.02 to -10.84 | Yes | **** | <0.0001 | 19.73 | 24 |
| Bone | NC - VHD | 6.528 | 24.22 | -17.69 | 0.9983 | -20.78 to -14.61 | Yes | **** | <0.0001 | 25.07 | 24 |
| Bone | PC - LD | 27.07 | 20.12 | 6.958 | 0.9983 | 3.871 to 10.04 | Yes | **** | <0.0001 | 9.857 | 24 |
| Bone | PC - MD | 27.07 | 18.7 | 8.376 | 0.9983 | 5.289 to 11.46 | Yes | **** | <0.0001 | 11.87 | 24 |
| Bone | PC - HD | 27.07 | 20.46 | 6.616 | 0.9983 | 3.529 to 9.703 | Yes | **** | <0.0001 | 9.372 | 24 |
| Bone | PC - VHD | 27.07 | 24.22 | 2.852 | 0.9983 | -0.2347 to 5.939 | No | ns | 0.0819 | 4.04 | 24 |
| Bone | LD - MD | 20.12 | 18.7 | 1.418 | 0.9983 | -1.669 to 4.505 | No | ns | 0.7148 | 2.009 | 24 |
| Bone | LD - HD | 20.12 | 20.46 | -0.342 | 0.9983 | -3.429 to 2.745 | No | ns | 0.9993 | 0.4845 | 24 |
| Bone | LD - VHD | 20.12 | 24.22 | -4.106 | 0.9983 | -7.193 to -1.019 | Yes | ** | 0.0047 | 5.817 | 24 |
| Bone | MD - HD | 18.7 | 20.46 | -1.76 | 0.9983 | -4.847 to 1.327 | No | ns | 0.5065 | 2.493 | 24 |
| Bone | MD - VHD | 18.7 | 24.22 | -5.524 | 0.9983 | -8.611 to -2.437 | Yes | *** | 0.0001 | 7.825 | 24 |
| Bone | HD - VHD | 20.46 | 24.22 | -3.764 | 0.9983 | -6.851 to -0.6773 | Yes | * | 0.0107 | 5.332 | 24 |

**Table S12.** Pairwise comparative statistical analyses of lead (Pb) concentrations across various rat organs under different treatment groups. Asterisks (*) indicate statistically significant differences at *p* < 0.05, while values with ns denote non-significant difference.
